# The Prediction of Steel Bar Corrosion Based on BP Neural Networks or Multivariable Gray Models

**DOI:** 10.1155/2023/2695142

**Published:** 2023-01-05

**Authors:** Juan Liu, Xuewei Bai

**Affiliations:** ^1^School of Civil Engineering, Liaoning Petrochemical University, Fushun 113001, China; ^2^College of Engineering, Shenyang Agricultural University, Shenyang 110866, China

## Abstract

The corrosion of steel bars in concrete has a significant impact on the durability of constructed structures. Based on the gray relational analysis (GRA) of the accelerated corrosion data and practical engineering data using MATLAB, a back propagation neural network (BPNN) model, a multivariable gray prediction model (GM (1, N)), and an optimization multivariable gray prediction model (OGM (1, N)) of steel corrosion were established by using a sequence of the key affecting factors. By comparing the prediction results of the three models, it is found that the GM (1, N) model has larger fitting and prediction errors for steel corrosion, while the OGM (1, N) model has smaller prediction errors in the accelerated corrosion data; the BPNN model offers more accurate predictions of the practical engineering data. The results show that the BPNN and OGM (1, N) models are all suitable for the prediction of steel bar corrosion in concrete structures.

## 1. Introduction

The corrosion of steel bars induces corrosion cracks in concrete structures. The appearance of cracks makes it easier for the corrosive media (H_2_O and O_2_) to reach the surface of the steel bars, which accelerates the corrosion rate of the steel bars. Corrosion reduces the cross section of steel bars and severely affects the bond strength between the steel bars and the concrete, resulting in structural failure. The reduced load capacity has a great impact on the durability and reliability of the structure [1]. It is difficult to measure the corrosion of steel bars in practice, especially those that are in service. In recent years, theoretical and empirical models have been proposed to estimate the extent of corrosion of steel bars after rust swelling and cracking of concrete structures. Bazant [2] and Zhang and Cheung [3] proposed a prediction model for the extent of steel corrosion according to its physical and chemical processes. Isgor and Razaqpur [4], Zhang et al. [5], Zheng et al. [6], and Xu et al. [7] conducted simulation tests in the laboratory to establish an empirical model of the corrosion rate of steel bars changing with environmental temperature, humidity, and other parameters. Based on a long-term exposure test and actual engineering durability test data, Guo et al. [8] proposed a formula for predicting the loss rate of steel bars. The theoretical model can reflect the physical and chemical processes of steel corrosion in concrete structures, and the influencing factors are comprehensive. However, many parameters in the model are difficult to determine. The empirical model can be closely linked with reality, but there are many complex factors in the model, which cannot be fully considered, resulting in certain inconsistencies with reality. Therefore, other methods are needed to predict the amount of steel corrosion.

As a method to determine whether or not variables are correlated and to determine the degree of their correlation, GRA provides a comprehensive assessment model. It was also applied to analyze the effects of the influencing factors on the steel corrosion which involves multiple variables with comprehensive correlations. Artificial neural network has been applied in the research of reinforcement corrosion [9, 10]. An et al. [11] combined the GRA and BPNN methods to predict the corrosion of steel bars; the results proved that this method can predict well. Luo et al. [12, 13] developed a hybrid enhanced Monte Carlo simulation and a dynamical adaptive enhanced simulation method coupled with support vector regression, which showed strong capability for application in the fatigue assessment of turbine bladed disks and structural reliability. Muiga et al. [14] adopted a gray prediction model (GM (1, 1)) to evaluate the carbonization of long-span reinforced concrete bridges. The accuracy of the prediction model was within a reasonable range and met the requirements of mathematical modeling. However, because of the complex corrosion mechanism of steel bars in concrete structures and the coupling relationship with crack width, protective layer thickness, and steel bar diameter, the GM (1, 1) model has a poor prediction effect sometimes, and the accuracy of its prediction is debatable [15]. In this study, a BPNN model, a multivariable gray model (GM (1, N)), and an optimized multivariable gray prediction model (OGM (1, N)) of steel corrosion are established by using the sequence of the key affecting factors after the gray relational analysis of the accelerated corrosion data. By comparing the calculation results of the three models with the practical engineering data, the applicability of the models is verified. The calculation results show that the BPNN and OGM (1, N) models perform well in terms of the prediction of the corrosion of steel bars in concrete structures and can provide some reference values for the evaluation of structural durability.

## 2. Prediction Methods

### 2.1. Gray Relational Analysis

Gray relational analysis refers to the degree of similarity between the curve geometry formed by the studied sequence and the change analysis of the influence factor sequence in the development process of the system. It helps to determine whether the connection is close by indicating the degree of connection between the curves. If changes in the trend of the two factors are consistent, the correlation between them will be greater; if the change in trend is inconsistent, the correlation will be lower [16, 17]. The gray correlation between sequences is reflected by the gray relational degree, which refers to the measurement of the correlation between the dependent variables over time or different objects, considering the relevancy between the factors so as to distinguish each factor. The greater the correlation between each influencing factor for the system, the closer relationship between them.

For a given system, assume that there are *N* variables:

(1) $$\begin{array}{c} X=\left\{X_{1},X_{2},\ldots ,X_{N}\right\}\text{.} \end{array}$$

With one out put *X*_1_ and *N* − 1 inputs *X*_*i*_(*i*=2,3,…, *N*). These two kinds of sequences have strong correlations with each other. For each variable *X*_*i*_(*i*=1,2,…, *N*), we assume that the sequence length is *n*, that is,

(2) $$\begin{array}{c} X_{i}=\left\{x_{i}\left(1\right),x_{i}\left(2\right),\ldots x_{i}\left(n\right)\right\},i=1,2,\cdots ,N\text{.} \end{array}$$

Data standardization is used to deal with the problems of inconsistent units among various sequence factors and inconsistent physical meanings. The mean and variance of each impact factor sequence are calculated as follows:

(3) $$\begin{array}{c} {\bar{x}}_{i}=\frac{1}{n}\overset{n}{\underset{k=1}{\sum }}x_{i}\left(k\right)\text{,}\\ \sigma_{i}=\sqrt{\frac{1}{n-1}\overset{n}{\underset{k=1}{\sum }}\left(x_{i}\left(k\right)-{\bar{x}}_{i}\right)},\left(i=2,3,\cdots ,N\right)\text{,} \end{array}$$

where ${\bar{x}}_{i}$ is the mean of each inputs sequence, and *σ*_*i*_ is the variance of each inputs sequence.

Then, we obtain

(4) $$\begin{array}{c} y_{i}\left(k\right)=\frac{\left(x_{i}\left(k\right)-{\bar{x}}_{i}\right)}{\sigma_{i}},i=2,3,\cdots ,N\text{,} \end{array}$$

where *y*_*i*_(*k*) is the i-th input sequence after normalization.

The difference sequences of the relevant factors are found as follows:

(5) $$\begin{array}{c} ∆y_{i}\left(k\right)=\left|y_{1}\left(k\right)-y_{i}\left(k\right)\right|,i=2,3,\cdots ,N\text{.} \end{array}$$

The correlation coefficient is calculated between the sequences as follows:

(6) $$\begin{array}{c} \lambda \left(x_{1}\left(k\right),x_{i}\left(k\right)\right)=\frac{\tau \max_{i}\max_{k}\left|x_{1}\left(k\right)-x_{i}\left(k\right)\right|+\min_{i}\min_{k}\left|x_{1}\left(k\right)-x_{i}\left(k\right)\right|}{\tau \max_{i}\max_{k}\left|x_{1}\left(k\right)-x_{i}\left(k\right)\right|+\Delta y_{i}\left(k\right)},0≺\tau ≺1\text{,} \end{array}$$

where *i*=2,3, ⋯, *N* and *k*=1,2, ⋯, *n* and *τ* is the resolution coefficient.

The gray relational degree was calculated as follows:

(7) $$\begin{array}{c} \lambda \left(X_{1},X_{i}\right)=\frac{1}{n}\overset{n}{\underset{k=1}{\sum }}\lambda \left(x_{1}\left(k\right),x_{i}\left(k\right)\right). \end{array}$$

The gray relational degrees of all the influencing factors were calculated and ranked the *λ*_*i*_ (*i*=2,3, ⋯, *N*) from high to low, as *λ*_*i*2_ > *λ*_*i*3_ > ⋯>*λ*_*iN*_, and the gray relational coefficient is between 0 and 1. The greater the relational degree, the stronger the relation, the correlation order is {*λ*_*i*2_, *λ*_*i*3_, ⋯, *λ*_*iN*_}. According to the correlation order, the standby schemes can be sorted and scientific foundations for decision-making are offered.

### 2.2. BP Neural Network

**Figure 1:**
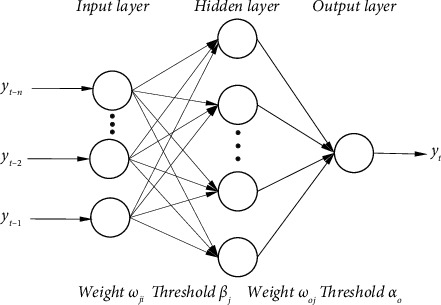
Structure of a BPNN with a single hidden layer [18].

The back propagation neural network (BPNN) can learn and store vast amounts of data because it is inspired by the structure of neurons, as illustrated in Figure 1 [18]. The BPNN is a feedforward multilevel neural network, which uses the network's adaptive mapping ability to carry out back propagation and is able to realize any non-linear operation from input to output [19, 20]. From the analysis of the network structure, the BPNN includes an input layer, an output layer, and a hidden layer (which can also be multiple layers). Variables are read from the input layer through the network's adaptive learning ability, and weights are calculated to determine the network output. The output result is compared with the target value, and the error is calculated. Through feedback and calculation for many iterations, the result can be output until the overall error of the network meets the requirements of the project.

The key to using the BPNN algorithm for fitting the amount of steel corrosion is to select the corrosion data set as the training set and to construct the mapping relationship between each corrosion value in the corrosion data set and the data of influential factors to allow effective training. Due to approximating ability to arbitrary nonlinear mapping, the BPNN has broad application in fitting steel corrosion.

#### 2.2.1. BP Neuron

Assuming that *x*_1_, *x*_2_, ⋯, *x*_*n*_ represents the input of the neurons 1,2, ⋯, *n*, respectively; *w*_*j*1_, *w*_*j*2_, ⋯, *w*_*jn*_ represent the connection weights of 1,2, ⋯, *n* and the *j-th* neuron, respectively; and *b*_*j*_ is the threshold.

Suppose the net input value *S*_*j*_ of the *j*th neuron is as follows:

(8) $$\begin{array}{c} S_{j}=\overset{n}{\underset{i=1}{\sum }}w_{ji}x_{i}+b_{j}=W_{j}X+b_{j}\text{,} \end{array}$$

where *X*=[*x*_1_, *x*_2_, ⋯,*x*_*n*_]^*T*^, *W*_*j*_=[*w*_*j*1_, *w*_*j*2_, ⋯, *w*_*jn*_]

If *x*_0_=1 and *w*_*j*0_=*b*_*j*_ we obtain *X*=[*x*_0_, *x*_1_, *x*_2_, ⋯,*x*_*n*_]^*T*^, *W*_*j*_=[*w*_*j*0_, *w*_*j*1_, *w*_*j*2_, ⋯, *w*_*jn*_]

Then,

(9) $$\begin{array}{c} S_{j}=\overset{n}{\underset{i=0}{\sum }}w_{ji}x_{i}=W_{j}X\text{.} \end{array}$$

After the net input *S*_*j*_ passes through the transfer function *f*(•) (this function is a monotonic rising function; there must be a maximum value), the output value *y*_*j*_ of the *j*-th neuron is obtained:

(10) $$\begin{array}{c} y_{j}=f\left(s_{j}\right)=f\left(\overset{n}{\underset{i=0}{\sum }}w_{ji}\cdot x_{i}\right)=F\left(W_{j}X\right)\text{.} \end{array}$$

#### 2.2.2. BP Network

Assuming that the input layer, hidden layer, and output layer of the BP network have *n*, *q*, and *m* nodes, respectively, the weight values between the input layer and hidden layer and that between the hidden layer and output layer are *v*_*ki*_ and *w*_*jk*_, respectively. The transfer function of the hidden layer is *f*_1_(•), then,

(11) $$\begin{array}{c} z_{k}=f_{1}\left(\overset{n}{\underset{i=0}{\sum }}v_{ki}x_{i}\right),k=1,2,\cdots ,q\text{.} \end{array}$$

The transfer function of the output layer is *f*_2_(•), and an output value is obtained in accordance with the group of weights and thresholds.

(12) $$\begin{array}{c} y_{j}=f_{2}\left(\overset{q}{\underset{k=0}{\sum }}w_{jk}z_{k}\right),j=1,2,\cdots ,m\text{.} \end{array}$$

#### 2.2.3. Error Back Propagation

In error back propagation, the output error of each layer of neurons is calculated through the output layer, the weight and bias value of the hidden layer of the grid are adjusted according to the error gradient descent method, and the parameters are continuously modified to reduce the error during the training process. The final error objective function is as follows:

(13) $$\begin{array}{c} E=\frac{1}{2}\overset{n}{\underset{j=1}{\sum }}\overset{m}{\underset{i=1}{\sum }}{\left(d_{i}-y_{i}\right)}^{2}\text{,} \end{array}$$

where *d*_*i*_ is the expected output value, and *E* is the error objective function.

### 2.3. Gray Model GM (1, N)

#### 2.3.1. Traditional Gray Model GM (1, N)

For a given system, assume that there are *N* variables:

(14) $$\begin{array}{c} X^{\left(0\right)}=\left\{X_{1}^{\left(0\right)},X_{2}^{\left(0\right)},\ldots ,X_{N}^{\left(0\right)}\right\}\text{.} \end{array}$$

With one out put *X*_1_^(0)^ and *N* − 1 inputs *X*_*i*_^(0)^(*i*=2,3,…, *N*). These two kinds of sequences have strong correlations with each other. For each variable *X*_*i*_^(0)^(*i*=1,2,…, *N*),we assume that the sequence length is *n*, that is,

(15) $$\begin{array}{c} X_{i}^{\left(0\right)}=\left\{x_{i}^{\left(0\right)}\left(1\right),x_{i}^{\left(0\right)}\left(2\right),\ldots x_{i}^{\left(0\right)}\left(n\right)\right\},i=1,2,\cdots ,N\text{.} \end{array}$$

The 1-AGO sequences of *X*_*i*_^(0)^ are defined as follows:

(16) $$\begin{array}{c} X_{i}^{\left(1\right)}=\left\{x_{i}^{\left(1\right)}\left(1\right),x_{i}^{\left(1\right)}\left(2\right),\ldots ,x_{i}^{\left(1\right)}\left(n\right)\right\},i=1,2,\cdots ,N\text{.} \end{array}$$

The mean sequences generated by consecutive neighbors of *X*_*i*_^(1)^ are defined as follows:

(17) $$\begin{array}{c} Z_{i}^{\left(1\right)}\left(k\right)=0.5\left(x_{i}^{\left(1\right)}\left(k-1\right)+x_{i}^{\left(1\right)}\left(k\right)\right),k=2,3,\cdots ,n,i=1,2,\cdots ,N\text{.} \end{array}$$

The expression of the GM (1, N) model is as follows [21]:

(18) $$\begin{array}{c} \frac{dx_{1}^{\left(1\right)}\left(t\right)}{dt}+ax_{1}^{\left(1\right)}\left(t\right)=\overset{N}{\underset{i=2}{\sum }}b_{i}x_{i}^{\left(1\right)}\left(t\right)\text{,} \end{array}$$

where *a* is the development coefficient of the sequence, ∑_*i*=2_^*N*^*b*_*i*_*x*_*i*_^(1)^(*t*) is the deriving term, and *b*_*i*_ is the driving coefficient.

The equation (18) can be regarded as a system of linear equations with respect to the parameters *p*=[*a*, *b*_2_, ...,*b*_*m*_]^*T*^, that is,

(19) $$\begin{array}{c} Bp=Y\text{,} \end{array}$$

where

(20) $$\begin{array}{c} \begin{array}{c} B=\left[\begin{array}{cccc} x_{2}^{\left(1\right)}\left(2\right) & \cdots & x_{m}^{\left(1\right)}\left(2\right) & -z_{1}^{\left(1\right)}\left(2\right)\\ x_{2}^{\left(1\right)}\left(3\right) & \cdots & x_{m}^{\left(1\right)}\left(3\right) & -z_{1}^{\left(1\right)}\left(3\right)\\ & \cdots & & \\ x_{2}^{\left(1\right)}\left(n\right) & \cdots & x_{m}^{\left(1\right)}\left(n\right) & -z_{1}^{\left(1\right)}\left(n\right) \end{array}\right],Y=\left[\begin{array}{c} x_{1}^{\left(0\right)}\left(2\right)\\ x_{1}^{\left(0\right)}\left(3\right)\\ \vdots \\ x_{1}^{\left(0\right)}\left(m\right) \end{array}\right] \end{array}\text{.} \end{array}$$

Using the ordinary least-squares estimate (OLSE) method, the parameters P can be obtained as follows:

(21) $$\begin{array}{c} p={\left[a,b_{2},\cdots ,b_{m}\right]}^{T}={\left(B^{T}B\right)}^{-1}B^{T}Y\text{.} \end{array}$$

Thus, the time response function of the GM(1,N) model can be derived as:

(22) $$\begin{array}{c} {\hat{x}}_{1}^{\left(1\right)}\left(t+1\right)=\left[x_{1}^{\left(0\right)}\left(1\right)-\frac{1}{a}\overset{m}{\underset{i=2}{\sum }}b_{i}x_{i}^{\left(1\right)}\left(t+1\right)\right]e^{-at}+\frac{1}{a}\overset{N}{\underset{i=2}{\sum }}b_{i}x_{i}^{\left(1\right)}\left(t+1\right)\text{.} \end{array}$$

It can get the predicted value as follows:

(23) $$\begin{array}{c} {\hat{x}}_{1}^{\left(0\right)}\left(t+1\right)={\hat{x}}_{1}^{\left(1\right)}\left(t+1\right)-{\hat{x}}_{1}^{\left(1\right)}\left(t\right)\text{.} \end{array}$$

From the above discussion, we can see that the GM (1, N) model has some obvious defects, such as the mean coefficient of the sequence in equation (18) and the driving term ∑_*i*=1_^*N*^*b*_*i*_*x*_*i*_^(1)^(*t*) in equation (18) are all constants, which may lead to poor predictive precision.

#### 2.3.2. OGM (1, N) Model

Zhai et al. [21] and Kaki et al. [22] proposed an optimized multivariable gray prediction model OGM (1, N), which is different from the traditional gray model GM (1, N). The calculation steps are as follows:

Let the two variable sequences *X*_1_^(0)^ and *X*_*i*_^(0)^(*i*=2,3,…, *N*) be defined as in equations (15) and (16). Whereas the 1-AGO sequences be defined as:

(24) $$\begin{array}{c} Z_{i}^{\left(1\right)}\left(k\right)=\left(\gamma -1\right)\left[x_{i}^{\left(1\right)}\left(k-1\right)\right]-\gamma \left[x_{i}^{\left(1\right)}\left(k\right)\right],k=2,3,\cdots ,n,i=1,2,\cdots ,N\text{,} \end{array}$$

where, the parameter *γ* can be adjusted according to the simulation accuracy.

Assume that the *X*_1_^(1)^ sequence approximates the exponential change law, and its influence factor sequence is *X*_2_^(1)^, *X*_3_^(1)^, ⋯, *X*_*m*_^(1)^; then, the *X*_*i*_^(1)^ sequence should satisfy the following first-order linear differential equation:

(25) $$\begin{array}{c} \frac{dX_{1}^{\left(1\right)}}{dt}=ax_{1}^{\left(1\right)}+\rho_{2}x_{2}^{\left(1\right)}+\rho_{3}x_{3}^{\left(1\right)}+\cdots +\rho_{m}x_{m}^{\left(1\right)}\text{.} \end{array}$$

The above formula is discretized, and a linear correction term *c*(*k* − 1) is added; then, the relationship between data points changes in the dependent variable sequence *ϕ*_*i*_. Subsequently, the differential equation expression of OGM(1, *N*) is obtained as follows:

(26) $$\begin{array}{c} x_{1}^{\left(0\right)}\left(k\right)+az_{1}^{\left(1\right)}\left(k\right)=\overset{n}{\underset{i=2}{\sum }}\rho_{i}x_{i}^{\left(1\right)}\left(k\right)+c\left(k-1\right)+\phi \text{.} \end{array}$$

Upon discretizing it, we obtain

(27) $$\begin{array}{c} x_{1}^{\left(0\right)}\left(k\right)+\gamma a\left[x_{1}^{\left(1\right)}\left(k-1\right)+x_{1}^{\left(1\right)}\left(k\right)\right]=\rho_{2}x_{2}^{\left(1\right)}\left(k\right)+\rho_{3}x_{3}^{\left(1\right)}\left(k\right)+\cdots +\rho_{m}x_{m}^{\left(1\right)}\left(k\right)+c\left(k-1\right)+\phi \text{,} \end{array}$$

where *k*=1,2, ⋯, *n*, and *c*(*k* − 1) reflects the linear relationship between the dependent variable and the independent variable.

Compared with the traditional gray model GM (1, N), an additional linear correction term *c*(*k* − 1) is introduced in (27) to improve the structure of the OGM (1, N) model.

In the new OGM (1, N) model (27), *N*+2 parameters, i.e., *P*=[*a*, *ρ*_2_, ⋯,*ρ*_*n*_, *c*, *ϕ*]^*T*^ need to be estimated. These parameters can be estimated by solving the system of linear equations:

(28) $$\begin{array}{c} Bp=Y\text{,} \end{array}$$

where:

(29) $$\begin{array}{c} B=\left[\begin{array}{cccccc} x_{2}^{\left(1\right)}\left(2\right) & \cdots & x_{m}^{\left(1\right)}\left(2\right) & -z_{1}^{\left(1\right)}\left(2\right) & 2 & 1\\ x_{2}^{\left(1\right)}\left(3\right) & \cdots & x_{m}^{\left(1\right)}\left(3\right) & -z_{1}^{\left(1\right)}\left(3\right) & 3 & 1\\ & \cdots & & & & \\ x_{2}^{\left(1\right)}\left(n\right) & \cdots & x_{m}^{\left(1\right)}\left(n\right) & -z_{1}^{\left(1\right)}\left(n\right) & n & 1 \end{array}\right],Y=\left[\begin{array}{c} x_{1}^{\left(0\right)}\left(2\right)\\ x_{1}^{\left(0\right)}\left(3\right)\\ \vdots \\ x_{1}^{\left(0\right)}\left(m\right) \end{array}\right]\text{,} \end{array}$$

(30) $$\begin{array}{c} P={\left[a\rho_{2}\cdots \rho_{n}c\phi \right]}^{T}={\left(B^{T}B\right)}^{-1}B^{T}Y\text{.} \end{array}$$

Substituting (30) into (27), the time response function of the GM (1, N) model can be derived as follows:

(31) $$\begin{array}{c} {\hat{x}}_{1}^{\left(1\right)}\left(k\right)=\overset{k-1}{\underset{t=1}{\sum }}\left[\eta_{1}\overset{n}{\underset{i=2}{\sum }}\eta_{2}^{t-1}\rho_{i}x_{i}^{\left(1\right)}\left(k-t+1\right)\right]+\eta_{2}^{k-1}{\hat{x}}_{1}^{\left(1\right)}\left(1\right)+\overset{k-2}{\underset{j=0}{\sum }}\eta_{2}^{j}\left[\left(k-j\right)\eta_{3}+\eta_{4}\right],k=1,2,\cdots ,n\text{,} \end{array}$$

where

(32) $$\begin{array}{c} \eta_{1}=\frac{1}{1+\gamma a}\text{,}\\ \eta_{2}=\frac{1-a}{1+\gamma a}\text{,}\\ \eta_{3}=\frac{c}{1+\gamma a}\text{,}\\ \eta_{4}=\frac{\phi }{1+\gamma a}\text{,}\\ {\hat{x}}_{1}^{\left(1\right)}\left(1\right)=x_{1}^{\left(0\right)}\left(1\right)\text{.} \end{array}$$

Which is referred to as the OGM(1, N) model.

It can get the predicted value as follows:

(33) $$\begin{array}{c} {\hat{x}}_{1}^{\left(0\right)}\left(k\right)={\hat{x}}_{1}^{\left(1\right)}\left(k\right)-{\hat{x}}_{1}^{\left(1\right)}\left(k-1\right),k=1,2,\cdots ,n\text{.} \end{array}$$

## 3. Results

### 3.1. Calculation of Gray Relational Degree

The calculation of the gray relational degrees is based on the corrosion test data of steel bars presented in Tables 1 and 2. The data in Table 1 is the accelerated corrosion data of reinforced concrete indoors collected by Liu and Wan [23]; and the data in Table 2 is the practical engineering data collected by Chen et al. [9].

In Table 1, the steel bar corrosion rate *ξ* and the steel bar corrosion extent *η* are included in the main sequence. The crack width *ω*, corrosion time *t*, corrosion current *i*, protective layer thickness *c*, steel bar diameter *d*, and steel bar spacing *D* are included in the reference sequence for calculating steel bar corrosion rate. The gray relational analysis of all influencing factors is carried out, and the gray relational degrees is obtained as follows:

(34) $$\begin{array}{c} \lambda_{i}=\left(\lambda_{\omega },\lambda_{t},\lambda_{i},\lambda_{c},\lambda_{d},\lambda_{D}\right)=\left(0.7704,0.7516,0.6268,0.6224,0.6021,0.5928\right)\text{.} \end{array}$$

Table 2 shows the crack width *ω*, concrete strength grade *f*_*cu*_, and steel bar diameter *d*. The thickness of the protective layer *c* serves as the reference sequence for the amount of steel corrosion. The gray correlation analysis of all influencing factors is carried out, and the gray relational degrees is obtained as follows:

(35) $$\begin{array}{c} \lambda_{i}=\left(\lambda_{d},\lambda_{f_{cu}},\lambda_{c},\lambda_{\omega }\right)=\left(0.7943,0.7648,0.7604,0.6783\right)\text{.} \end{array}$$

### 3.2. Establishment of Prediction Models Based on BPNN, GM (1, N), and OGM (1, N) Models

Using the accelerated corrosion data in Table 1, according to the results of the correlation analysis, the BPNN model selects the crack width, corrosion time, corrosion current, and protective layer thickness to form the input vector; the steel corrosion rate forms the output vector. A BPNN with four nodes in the input layer and one node in the output layer is established. Based on multiple fitting trials, the hidden layer is set to eight layers, and the learning rate is 0.035. In order to prevent the network from over-fitting, the noise intensity is set as 0.01. The target error value is specified as 0.65 × 10^−3^.

In GM (1, N) and OGM (1, N) modeling, crack width, corrosion time, and corrosion current with a greater correlation were included in the correlation sequences, and GM (1, 3) and OGM (1,3) models were established. The GM (1, 3) model predicted the parameters to be *G*=[*a*, *ρ*_2_, ⋯,*ρ*_*n*_]^*T*^=[−0.1001,1.2176*e*^−6^, 0.0272,0.0298]^*T*^. After repeated trials, when *γ*=0.05, the fitting and prediction results of the OGM (1, 3) model were ideal, and the calculated prediction parameters are as follows:

(36) $$\begin{array}{c} G={\left[a,\rho_{2},\cdots ,\rho_{n},c,\phi \right]}^{T}={\left[0.0513,0,0.0332,0.0103,0.001,0.0037\right]}^{T}\text{,}\\ \eta_{1}=1.0026\text{,}\\ \eta_{2}=1.0515\text{,}\\ \eta_{3}=0.001\text{,}\\ \eta_{4}=0.0037\text{.} \end{array}$$

According to practical engineering data presented in Table 2, gray relational degrees is relatively high; therefore, both the BPNN and gray multivariable model choose all influencing factors for modeling. The BPNN modeling process is the same as that using Table 1. The predicted parameters obtained by calculation using the GM (1, 4) model are *G*=[*aρ*_2_ ⋯ *ρ*_*n*_]^*T*^=[0.0643, −8.3266,0.5578, −0.2714,0.0427]^*T*^. After repeated trials, when *γ*=0.75, the simulation and prediction results of the OGM (1, 4) model were satisfactory. The calculated prediction parameters are as follows:

(37) $$\begin{array}{c} G={\left[a,\rho_{2},\cdots ,\rho_{n},c,\phi \right]}^{T}={\left[-4.0305,0.1797,-0.0306,-0.0849,-0.0112,0.6459,15.7473\right]}^{T}\\ \eta_{1}=1.0085\text{,}\\ \eta_{2}=1.0113\text{,}\\ \eta_{3}=0.6514\text{,}\\ \eta_{4}=15.8810\text{.} \end{array}$$

### 3.3. Comparison of Calculation Results

**Figure 2:**
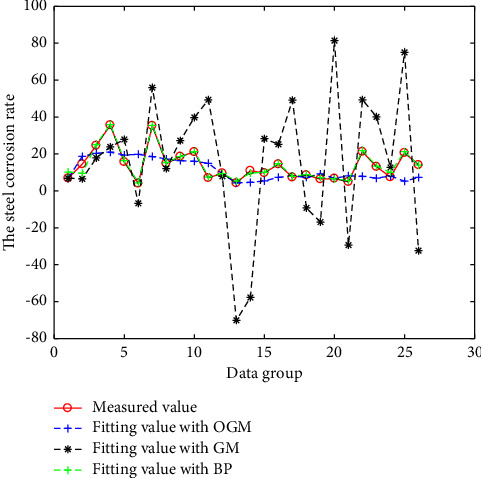
Results of the different models for rapid corrosion test.

**Figure 3:**
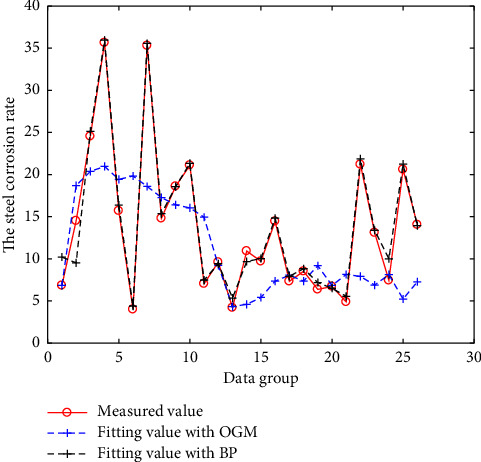
Results of the BPNN and OGM (1, N) models for rapid corrosion test.

**Figure 4:**
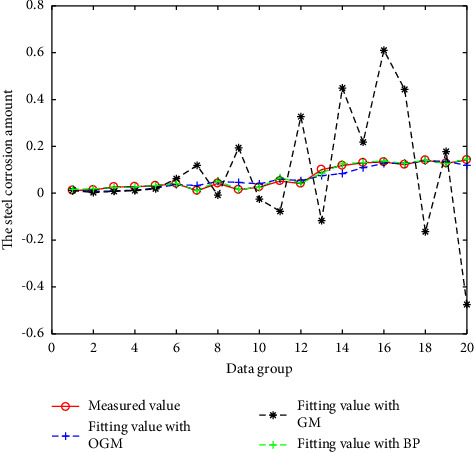
Results of the different models for project data.

**Figure 5:**
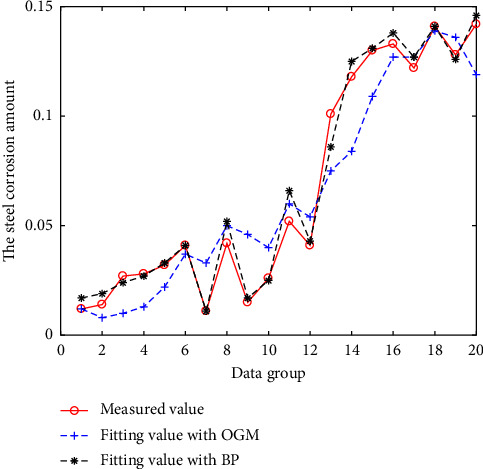
Results of the BPNN and OGM (1, N) models for project data.

The above three models are all modeled and fitted with N-3 groups of data, and the remaining 3 groups of data are predicted. The fitting results are shown in Figures 2–5. And the prediction results and errors of the different models are listed in Tables 3 and 4.

## 4. Discussion

It can be observed from Figure 2, for the corrosion data of the accelerated corrosion data, among those of the three calculation methods, the fitting error of the GM (1, N) model is relatively large, while the other two models have relatively small errors.

It can be seen from Figure 3 that the fitting error of the BPNN is much smaller than that of OGM (1, N). Because steel corrosion is affected by many factors, there is a highly nonlinear relationship between steel corrosion and each influencing factor, and the available data is limited. It is especially suitable for BPNN fitting; however, the BPNN may also exhibit over-fitting, resulting in regularity distortion and the predicted value error being relatively large.

It can be seen from Figures 4 and 5, for the measured corrosion data of practical engineering buildings, due to the large discretization of the measured values, the fitting and prediction errors obtained using the GM (1, N) and OGM (1, N) models are both large, while the predicted results obtained using the BPNN model are close to the real values.

It can be seen from the comparative analysis of the prediction results in Tables 3 and 4, the average fitting errors of the OGM (1, N) model for both the fast test and the engineering scatter data are larger than those of the BPNN model, whereas the predicted value of the OGM (1, N) for the accelerated corrosion data is closer to the real value with minimal error. However, the BPNN model has good prediction precision for practical engineering data.

## 5. Conclusions

The corrosion of steel bars has a great influence on the safety and durability of reinforced concrete structures. There are many factors that influence the corrosion of steel bars in practical engineering, such as the crack width of the concrete structure, strength grade of concrete, diameter of steel bar, thickness of the concrete protective layer, and environmental factors of the project. In this paper, the BPNN, GM (1, N), and OGM (1, N) are used to fit and predict the accelerated corrosion data and practical engineering data of the concrete structure, and the following conclusions are drawn:

1. Compared with the traditional gray model GM (1, N), the OGM (1, N) model exhibits a higher fitting and predicting accuracy.
2. Compared with the GM (1, N) and OGM (1, N) models, the BPNN model exhibits a higher fitting accuracy for the two kinds of data (the accelerated corrosion data and practical engineering data
3. The BPNN has a higher prediction accuracy for the practical engineering data, while the OGM (1, N) model has a higher prediction accuracy than the BPNN for the accelerated corrosion data.
4. In the practical engineering data, there are several influencing factors for the corrosion of steel bars. In this study, we only analyze a few influencing factors that cause greater correlation and do not consider the atmospheric parameters of the service environment of the actual engineering structure. More relevant data can be collected for model forecasting in actual engineering applications [23].

## Figures and Tables

**Table 1:** Results of indoor accelerated corrosion test of reinforced concrete.

Data group	*ξ* (mm/a)	*t* (min)	*D* (mm)	*c* (mm)	*ω* (mm)	*i* (mm)	*d* (mm)
1	0.012	2185	90.4	24.9	0.08	0.078	12.06
2	0.014	2985	90.4	24.9	0.13	0.078	12.12
3	0.027	4185	90.4	24.9	0.15	0.078	12.31
4	0.028	4985	90.4	24.9	0.21	0.078	12.33
5	0.032	5885	90.4	24.9	0.45	0.078	12.36
6	0.041	6655	90.4	24.9	0.62	0.078	12.39
7	0.011	6655	88	44	0.06	0.018	12.1
8	0.042	7815	90.4	24.9	0.71	0.078	12.4
9	0.015	7815	88	44	0.07	0.018	12.15
10	0.026	9134	88	44	0.08	0.018	12.3
11	0.052	9185	90.4	24.9	0.83	0.078	12.43
12	0.041	11254	88	44	0.14	0.018	12.39
13	0.101	11295	90.4	24.9	0.93	0.078	12.5
14	0.118	21735	90.4	24.9	0.98	0.078	12.63
15	0.13	21735	88	44	1.42	0.018	12.76
16	0.133	32300	88	44	1.61	0.018	12.87
17	0.122	32925	90.4	24.9	1.01	0.078	12.65
18	0.141	38131	88	44	1.62	0.018	13.01
19	0.128	38135	90.4	24.9	1.13	0.078	12.68
20	0.142	61385	90.4	24.9	1.62	0.078	13.08
21	0.148	61385	88	44	2.91	0.018	13.25
22	0.163	87645	90.4	24.9	3.69	0.078	13.65
23	0.173	87645	88	44	3.24	0.018	13.71

**Table 2:** The practical engineering data (Liaoning Benxi and Shanxi steel plant).

Data group	*η* (mm)	*ω* (mm)	*f*_*cu*_ (MPa)	*c* (mm)	*d* (mm)
1	6.84	0.18	21.21	15	22
2	14.51	0.25	21.63	17	20
3	24.53	0.333	26.58	30	18
4	35.64	0.667	29.86	30	22
5	15.7	0.5	22.34	55	30
6	4	0.75	31.76	14	30
7	35.3	0.667	21.21	30	25
8	14.8	1.1	25.68	15	20
9	18.6	0.667	20.31	12	25
10	21.1	0.667	23.88	16	25
11	7.04	0.85	18	17.3	12
12	9.6	2	18	15.4	12
13	4.2	1.8	18	12.1	12
14	10.92	0.5	18	14.8	12
15	9.7	0.5	20	20	8
16	14.45	0.225	20	20	8
17	7.33	0.3	20	40	12
18	8.52	0.35	10	20	12
19	6.37	0.25	20	10	12
20	6.72	1.15	18	15.7	12
21	4.88	0.3	20	20	12
22	21.2	0.7	28.21	30	25
23	13.1	0.5	22.31	33.5	30
24	7.44	0.2	21.21	20	20
25	20.6	1	21.64	30	28
26	14.05	0.6	30.54	18	12
27	7.68	0.9	18	13.1	12
28	8.32	1.5	18	11.7	12
29	5.85	0.3	20	20	16

**Table 3:** Comparison of the average fitting errors between models.

	BPNN (%)	GM (1, N) (%)	OGM (1, N) (%)
Accelerated corrosion test data	9.37	87.3	15.48
Project data	10.30	94.21	49.98

**Table 4:** Comparison of prediction values and errors between models.

	Actual values	BPNN	GM (1, N)	OGM (1, N)
Accelerated corrosion test data	0.148	0.151	−1.59	0.143
	0.163	0.147	0.386	0.162
	0.173	0.1556	0.157	0.17

The average prediction error		7.29%	>100%	1.9%

Project data	7.68	8.381	−32.395	5.998
	8.32	7.943	103.206	2.314
	5.85	8.78	0.996	5.21

The average prediction error		8.2%	>100%	48%

## Data Availability

The indoor accelerated corrosion test of reinforced concrete data and actual engineering test data used to support the findings of this study are included within the articles “Predict Corrosion Degree of Steel Bars in Reinforcing Concrete Based on ABC-BP Neural Network” and “Assessment on corrosive degree of reinforcement in concrete by artificial neural networks,” respectively.
